# Exercise-induced signaling pathways to counteracting cardiac apoptotic processes

**DOI:** 10.3389/fcell.2022.950927

**Published:** 2022-08-11

**Authors:** Hamed Alizadeh Pahlavani

**Affiliations:** Department of Physical Education, Farhangian University, Tehran, Iran

**Keywords:** exercise, apoptotic, cardiovascular diseases, cardiomyocytes, signaling, signaling pathways

## Abstract

Cardiovascular diseases are the most common cause of death in the world. One of the major causes of cardiac death is excessive apoptosis. However, multiple pathways through moderate exercise can reduce myocardial apoptosis. After moderate exercise, the expression of anti-apoptotic proteins such as IGF-1, IGF-1R, p-PI3K, p-Akt, ERK-1/2, SIRT3, PGC-1α, and Bcl-2 increases in the heart. While apoptotic proteins such as PTEN, PHLPP-1, GSK-3, JNK, P38MAPK, and FOXO are reduced in the heart. Exercise-induced mechanical stress activates the β and α5 integrins and subsequently, focal adhesion kinase phosphorylation activates the Akt/mTORC1 and ERK-1/2 pathways, leading to an anti-apoptotic response. One of the reasons for the decrease in exercise-induced apoptosis is the decrease in Fas-ligand protein, Fas-death receptor, TNF-α receptor, Fas-associated death domain (FADD), caspase-8, and caspase-3. In addition, after exercise mitochondrial-dependent apoptotic factors such as Bid, t-Bid, Bad, p-Bad, Bak, cytochrome c, and caspase-9 are reduced. These changes lead to a reduction in oxidative damage, a reduction in infarct size, a reduction in cardiac apoptosis, and an increase in myocardial function. After exercising in the heart, the levels of RhoA, ROCK1, Rac1, and ROCK2 decrease, while the levels of PKCε, PKCδ, and PKCɑ are activated to regulate calcium and prevent mPTP perforation. Exercise has an anti-apoptotic effect on heart failure by increasing the PKA-Akt-eNOS and FSTL1-USP10-Notch1 pathways, reducing the negative effects of CaMKIIδ, and increasing the calcineurin/NFAT pathway. Exercise plays a protective role in the heart by increasing HSP20, HSP27, HSP40, HSP70, HSP72, and HSP90 along with increasing JAK2 and STAT3 phosphorylation. However, research on exercise and factors such as Pim-1, Notch, and FAK in cardiac apoptosis is scarce, so further research is needed. Future research is recommended to discover more anti-apoptotic pathways. It is also recommended to study the synergistic effect of exercise with gene therapy, dietary supplements, and cell therapy for future research.

## Introduction

**FIGURE 1:**
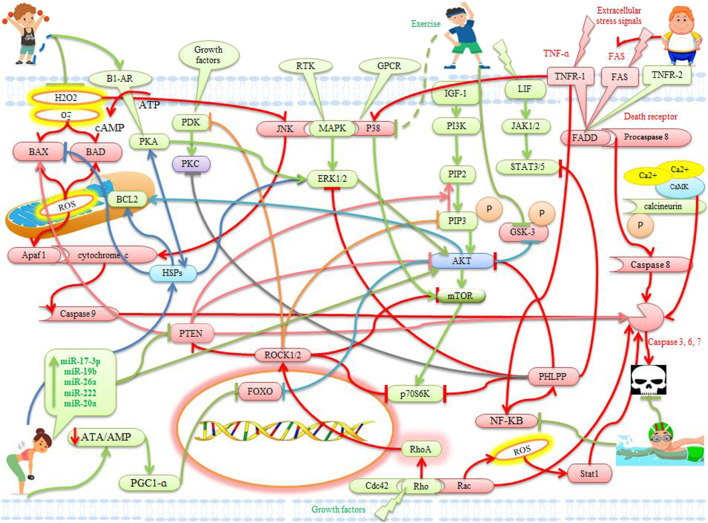
The effects of exercise on apoptotic and anti-apoptotic pathways in the heart. Description is available in the text.

Cardiovascular diseases (CVDs) are the most common cause of death in many parts of the world. If this condition persists, cardiac mortality will increase in the future (Alizadeh Pahavani et al., 2020). Hypoxia due to atherosclerosis is one of the causes of damage to heart muscle cells and leads to the programmed death of cardiomyocytes called apoptosis (Pahlavani and Veisi, 2018). Apoptosis occurs when cardiomyocytes are exposed to hydrogen peroxide (H_2_O_2_) or superoxide anion ( ${\text{O}}_{2}^{-}$ ) (Von Harsdorf et al., 1999). Apoptosis as a physiological program of cell death is involved in many heart disorders (Huang et al., 2012). Apoptosis plays an important role in myocardial loss after myocardial infarction, ischemia/reperfusion (I/R) injury, and myocardial ischemia. Excessive apoptosis participates in the subsequent process of regeneration of the left ventricle and the development of heart failure (Teringova and Tousek, 2017; Dong et al., 2019; Gill et al., 2002). Apoptosis is initiated and performed through two main pathways, intracellular and extracellular (Xia et al., 2016). The intracellular apoptotic pathway is called the mitochondrial apoptotic pathway, which is stimulated by oxidative stress, calcium overload, and DNA damage, leading to mitochondrial permeability and cytochrome C release (Xia et al., 2016). Cytosolic cytochrome c and the apoptosis protease activating factor 1 (Apaf-1) then cause the formation of apoptosome and activated caspase-9 (Xia et al., 2016) (Figure 1). In contrast, extracellular apoptosis is initiated by extracellular stress signals such as tumor necrosis factor-α (TNF-α), Fas ligand (FasL), and TNF-related apoptosis-inducing ligand (TRAIL), which bind to their unique death receptors such as TNF-α receptor 1 (TNFR1), Fas, and TRAIL receptor 1/2 (TRAILR1/2) (Xia et al., 2016). Death receptors then absorb the fas-associated death domain (FADD) and procaspase-8, leading to activation of caspase-8. The initiator caspases-9 or 8 activate executive caspases-3, 6, and 7, leading to apoptotic cell death (Xia et al., 2016) (Figure 1). However, apoptosis as a biological process can be modulated by genetic, pharmacological, or sports interventions (Xia et al., 2016). Of these, physical activity is one of the most important because aerobic exercise (70–75% VO_2max_) makes the heart resistant to myocardial infarction in rats (Alizadeh Pahavani et al., 2020). A scientific study of more than 3,000 people found that aerobic exercise reduced hospitalization due to heart failure as well as cardiovascular events (Mediano et al., 2018; Wang et al., 2021). Other studies have shown that aerobic exercise (Equivalent to 70–75% VO_2max_) increases anti-apoptotic factors such as B-cell lymphoma 2 (Bcl-2) and decreases apoptotic factors such as BCL2 associated X (Bax) in the myocardium. There is also evidence that increased phosphorylation of protein kinase B (PKB), also known as Akt, one of the upstream proteins in the Bcl-2 family, improves heart survival through exercise. Bcl-2 and phosphorylated-BAD then inhibit apoptotic activity (Huang et al., 2012). In ovariectomized rats, swimming training induces the expression of Bcl-2 and Mir-133 in the myocardium and then prevents myocardial apoptosis (Habibi et al., 2016). Mice 18 months after myocardial infarction has been reported to significantly increase left ventricular function and survival through swimming practice (5 times a week for 8 weeks) as well as suppressing myocardial fibrosis and apoptosis (Zhao et al., 2018). Hence, intervention in exercise-induced apoptotic pathways and cell survival may be a promising treatment strategy for diseases and cardiovascular disorders such as myocardial infarction, ischemic/reperfusion (I/R) injury, chemotherapy, and heart failure. To identify new pathways and their effects on each other, a comprehensive understanding of apoptotic pathways and cell survival is essential. This study aims to investigate the effect of exercise-related anti-apoptotic pathways on apoptotic pathways and cell survival to modulate cardiomyocytes survival; Because modulating apoptosis and preventing excessive apoptosis is a useful treatment strategy for cardiovascular disease.

## Extracellular apoptosis signaling pathways

### TNF-α/NF-κB pathway

Tumor necrosis factor-α (TNF-α) is secreted by mast cells and macrophages, which is dependent on oxidative stress. Global and persistent TNF-α deficiency reduces inflammatory cells and cardiac apoptosis after permanent ischemia or I/R injury. In addition, deletion of TNF-α receptor 1 (TNFR1), but not TNFR2, protects the heart against I/R damage, suggesting that the harmful effect of TNF-α may be mediated by TNFR1. Studies have shown that deletion of TNFR1 reduces apoptosis and suppresses NF-κB, p38, and JNK2 activation, while deletion of TNFR2 increases apoptosis and activation of NF-κB and p38. Activation of TNFR1 and TNFR2 mediates harmful and beneficial signals, respectively (Xia et al., 2016) (Figure 1). In rats short-term (3 days) and long-term exercise (3 weeks) can reduce myocardial apoptosis by reducing TNF-α, caspase-8, and caspase-3. While long-term exercise is more effective than short-term exercise (Meng et al., 2017). There is evidence that in rats apoptotic cells are lower in the exercise group (48–66% of VO_2max_ for 12 weeks) (Qin, Dong, Wang, Xu, Wang, Qu, et al.) with high blood pressure than in the sedentary group. Under exercise conditions, decreased apoptosis is due to decreased Fas-dependent apoptotic pathway factors such as Fas-ligand protein, Fas death receptor, TNFR-1, Fas-associated death domain (FADD), caspase-8, and caspase-3. In addition, under exercise conditions, mitochondrial-dependent apoptotic pathway factors such as Bid, t-Bid, Bad, p-Bad, Bak, cytochrome c, caspase-9, and caspase-3 are reduced. In contrast, increased pathways supporting cardiac survival have been reported with protein levels of IGF-1, IGF-1R, p-PI3K, p-Akt, p-Bad, and Bcl2. These results show the new therapeutic effects of exercise on hearts with high blood pressure to prevent apoptosis (Huang et al., 2012) (Table 1). After I/R in rats, the exercise group (56–66% of VO_2max_ for 10 days) (Qin, Dong, Wang, Xu, Wang, Qu, et al.) compared to the control group decreases caspase-7 (15-fold), TNF-ɑ (2.5-fold), TNF-ɑ receptor (12-fold), and pro-apoptosis factors (Cradd, FADD, NF-KB). In general, depletion of these genes reduces myocardial apoptosis (Dickson et al., 2008) (Figure 1) (Table 1). In obese mice under exercise (48–66% of VO_2max_ for 3 months), compared to obese mice, levels of TNF-ɑ, Fas ligand, Fas receptors, FADD, caspase-8, and caspase-3 and protein levels of Bad, Bax, the Bax to Bcl2 ratio, caspase-9 is less. While extracellular and intracellular apoptosis pathways are more active in obese animals’ hearts. Therefore, exercise (4 weeks) can prevent extracellular and intracellular apoptotic pathways (Lee et al., 2013; Otaka et al., 2018) (Table 1). In rats, the exercise group (56–73 VO_2max_ for 10 weeks) compared with the group without ovaries decreases protein levels of TNF-α, TNFR-1, Fas-ligand protein, Fas receptors, FADD, caspase-8, and caspase-3, as well as *t*-Bid, Bad, Bak, Bax, cytosolic cytochrome c, and caspase-9. These findings could indicate a new therapeutic effect of exercise training to prevent cardiac apoptosis in postmenopausal women or oophorectomized (Huang et al., 2016) (Table 1). Possible beneficial mechanisms of exercise can be the reduction of oxidative stress and inflammatory factors due to exercise adaptation.

**TABLE 1:** A summary of exercise studies on the factors affecting cardiac apoptosis.

Type of exercise	Intensity and duration	Apoptotic and anti-apoptotic factors	Changes	Reference
Aerobic exercise	48–75% of VO2max for 12 weeks	IGF-1, IGF-1R, p-PI3K, p-Akt, p-Bad, Bcl2	Increase	Huang et al. (2012)
		Fas-ligand protein, Fas death receptor, TNFR-1, Fas-associated death domain (FADD), caspase-8, caspase-3, Bid, t-Bid, Bad, p-Bad, Bak, cytochrome c, caspase-9	Decrease	
Short-term and long-term exercise	—	TNF-α, caspase-8, caspase-3	Decrease	Meng et al. (2017)
Aerobic exercise	56–66% of VO2max for 10 days	caspase-7, TNF-ɑ, TNF-ɑ receptor, Cradd, FADD, NF-KB	Decrease	Dickson et al. (2008)
Aerobic exercise	48–66% of VO2max for 3 months	TNF-ɑ, Fas ligand, Fas receptors, FADD, caspase-8, caspase-3, Bad, Bax, the Bax to Bcl2 ratio, caspase-9	Decrease	(Lee et al., 2013; Otaka et al., 2018)
Aerobic exercise	56 to 73 VO2max for 10 weeks	TNF-α, TNFR-1, Fas-ligand protein, Fas receptors, FADD, caspase-8, caspase-3, t-Bid, Bad, Bak, Bax, cytochrome c, caspase-9	Decrease	Huang et al. (2016)
Aerobic exercise	40–60% of VO2max	IGF-1, Akt	Increase	(Kwak, 2013; Tao et al., 2015)
Swimming	8 weeks	IGF1R, Akt, Bcl-2	Increase	(Kwak, 2013; Lay et al., 2021)
Aerobic exercise	48 to 66 VO2max for 10 weeks	IGF-I, IGFI-R, PI3K, Akt	Increase	Cheng et al. (2013)
		Bcl-xL, p-BAD, caspase-3	Decrease	
Aerobic exercise	48% of VO2max for 6 weeks	FGF-21/FGFR1/PI3K/AKT	Increase	Bo et al. (2021a)
Voluntary exercise		IGF1/PI3K/AKT	Increase	Wang et al. (2021)
Voluntary moderate exercise	6 weeks	AKT, ERK-1/2	Increase	Chodari et al. (2016)
Aerobic exercise	48–66% of VO2max for 4 weeks	Akt, ERK-1/2, p70S6K, AMPK	Increase	Pons et al. (2013)
Endurance training	72% of VO2max for 12 weeks	ERK-1/2, ANP/BNP	Increase	Ho et al. (2012)
		cytochrome C, caspase-3	Decrease	
Aerobic exercise	40–85% of VO2max for 1 week	ERK-1/2	Increase	(Iemitsu et al., 2006; Budiono et al., 2012; Liao et al., 2019)
A training session	60–80% of VO2max	JNK, p38	Decrease	(Boluyt et al., 2003; Ludlow et al., 2017)
	48–60% of VO2max	p38		
Physical exercise	50–55% of VO2max for 2 months	P38, mTOR, P70S6k, 4EBP1	Increase	Pieri et al. (2014)
treadmill exercise	35–45% of VO2max	p38, JNK, NF-κB, caspase-1, IL-1β, IL-6, Bax, caspase-3, p53	Decreased	(Lagranha et al., 2007; Ko et al., 2013; Hong et al., 2017)

### IGF-1/PI3K/Akt pathway

**FIGURE 2:**
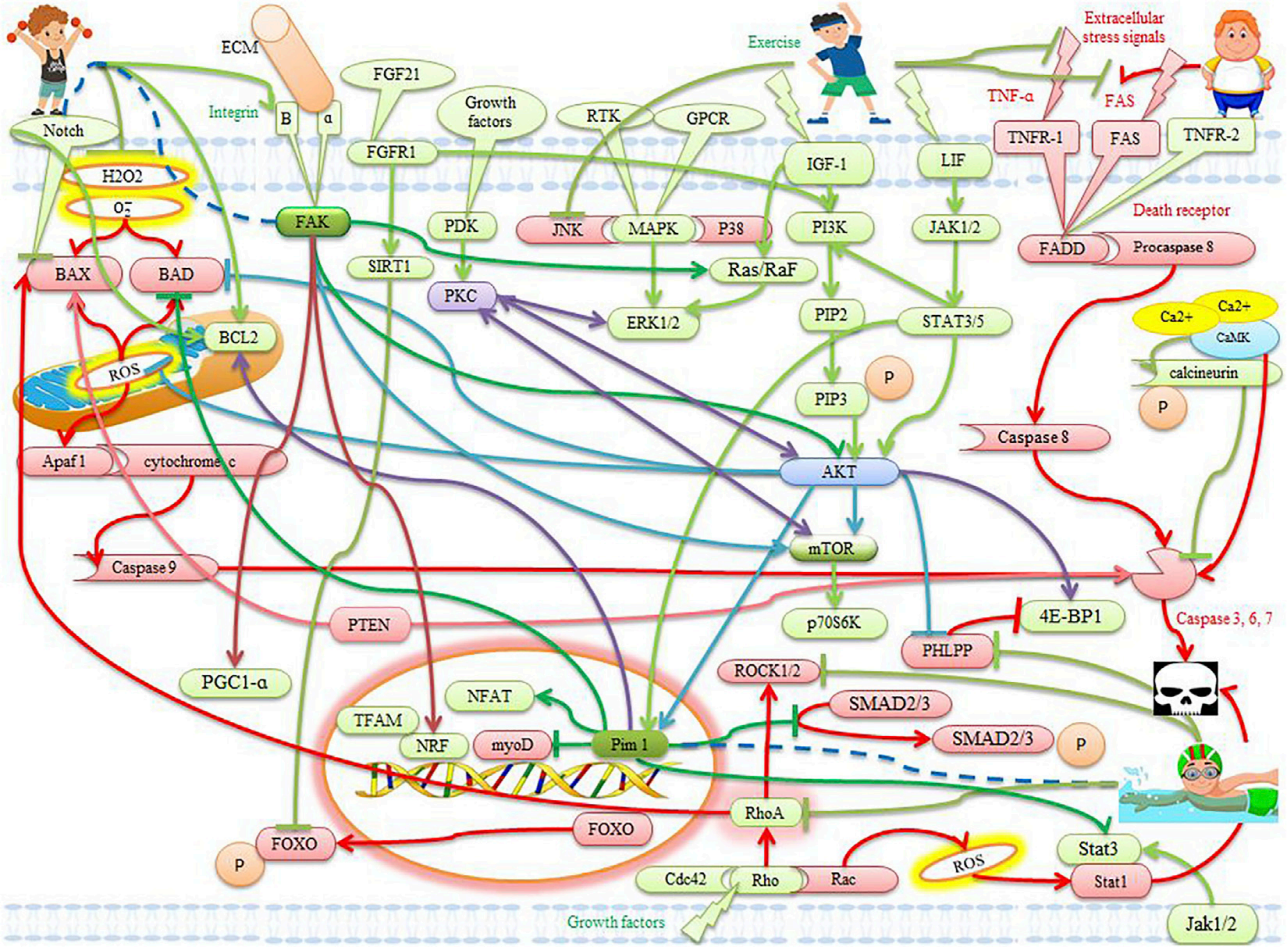
The effects of exercise on apoptotic and anti-apoptotic pathways in the heart. Description is available in the text.

The Insulin-like growth factor (IGF)-1/phosphoinositide 3-kinases (PI3K)/Akt signaling pathway is activated after stimulation of growth factors, cytokines, hormones, and muscle contractions (Xia et al., 2016). Akt has 3 isoforms called AKT1 and AKT2, AKT3 (Wang et al., 2021) (Table 1). Both Akt family members Akt1 and Akt2 are abundantly expressed in cardiomyocytes (Xia et al., 2016). Among these, AKT1 is known as an isoform-specific to physiological hypertrophy and exercise-induced heart growth (Wang et al., 2021). In transgenic mice, an 80% increase in AKT lead to a 2.2-fold increase in heart weight compared with controls (Wang et al., 2021). Protection of cardiomyocytes via the IGF-1/PI3k/Akt pathway against apoptosis is associated with the inactivation of Bad pro-apoptotic protein. It seems in mitochondria, Akt protects the integrity of the outer membrane (Tao et al., 2015; Xia et al., 2016). Nuclear overexpression of Akt protects cardiomyocyte apoptosis without phosphorylation of cytoplasmic Akt. Nuclear Akt targets in the myocardium include the forkhead box (FOX) transcription factor and serine/threonine kinase (Pim-1) (Xia et al., 2016) (Figures 1,2). In humans, exercise (40–60% of VO_2max_) increases cardiac expression of IGF-1 and Akt and leads to cardiac hypertrophy and survival (Kwak, 2013; Tao et al., 2015) (Table 1). In older rats, also the reduction in cardiac survival pathway proteins such as IGF1R, Akt, and Bcl-2 improves with exercise (swimming for 8 weeks) (Kwak, 2013; Lay et al., 2021) (Table 1). In addition, levels of IGF-1, IGF-1R, p-PI3K, p-Akt, and Bcl2 are higher in the exercise group (48–66% of VO_2max_ for 12 weeks) than in the sedentary group and the sedentary hypertensive group. As a result, exercise prevents intracellular apoptotic pathways in the hearts of patients with hypertension and increases cardiac survival pathways (Huang et al., 2012) (Table 1). It is reported that in the diabetic rat group the components of hypertrophy and cardiac survival pathways such as IGF-I, IGF-I receptor (IGFI-R), PI3K, Akt, and anti-apoptotic proteins such as Bcl-2, Bcl-xL, and p-BAD decrease significantly, while increases in the diabetic aerobic exercise group (48–66 VO_2max_ for 10 weeks). In addition, caspase-3, the abnormal structure of the myocardium, and an increase in cardiac apoptotic cells are more severe in the diabetic group than in the diabetic group with exercise. These findings emphasize the role of exercise in preventing myocardial apoptosis (Cheng et al., 2013). Aerobic exercise (48% of VO_2max_ for 6 weeks) also significantly reduces oxidative stress (H_2_O_2_) and apoptosis by activating the Fibroblast growth factor 21 (FGF-21)/FGFR1/PI3K/AKT pathway, which ultimately improves heart function in mice with myocardial infarction (Bo et al., 2021a) (Figure 1) (Table 1). Hence, other studies have suggested voluntary exercise such as running and swimming as a recommendation to prevent CVD because exercise via the IGF1/PI3K/AKT pathway improves heart function. This pathway can reduce heart damage in a variety of ways, such as cardiomyocyte proliferation and growth, physiological hypertrophy, increased cardiac apoptotic capacity, and inhibition of fibrosis (Wang et al., 2021) (Table 1). The possible mechanism of physiological cardiac hypertrophy in exercise is to increase the demand of cells and tissues of the heart for oxygen and blood supply. Hence the survival, growth and proliferation of heart cells and counteracting apoptosis seems reasonable during exercise.

### MAPKs pathway

Mitogen-activated protein kinases (MAPKs) regulate cell functions such as survival, growth, and differentiation in response to extracellular signals. Extracellular signals lead to phosphorylation and activation of factors such as extracellular signal-regulated kinase 1/2 (ERK-1/2 or p44/42), Jun N-terminal kinases (JNK), and p38 isoforms (Xia et al., 2016). Myocytes are more susceptible to apoptosis in ERK2-deficient mice, suggesting a role for ERK-1/2 in cell survival. However, continuous activation of ERK-1/2 and its nuclear transport contribute to cardiomyocyte apoptosis. In addition, ERK-1/2 is also activated by hydroxyl radicals via Ras/Raf-1 MAPKKK and has a protective role. Mice with cardiac Raf-1 deficiency show myocyte apoptosis at 3 weeks of age and heart failure late in life (Xia et al., 2016). Voluntary moderate exercise (6 weeks) increases the cardiac activity of AKT and ERK-1/2 in diabetic rats, which in turn leads to a reduction in cardiac apoptosis (Chodari et al., 2016). In wild-type mice, exercise (48–66% of VO_2max_ for 4 weeks) reduces the size of infarction by up to 60% by increasing the phosphorylation of kinases such as Akt, ERK-1/2, p70S6K, and AMPK (Pons et al., 2013) (Figure 1) (Table 1). In mice, excessive endurance training (72% of VO_2max_ for 12 weeks) also leads to increased myocyte cross-section, hypertrophy-related pathways (ERK-1/2), hypertrophic markers (ANP/BNP), and decreased pro-apoptotic molecules (cytochrome C and caspase-3) (Ho et al., 2012) (Table 1). It seems also exercise (40–85% of VO_2max_ for 1 week) can significantly improve cardiac ischemic tolerance by increasing ERK-1/2 and inhibiting GSK-3β phosphorylation in mice (Budiono et al., 2012). In mice, increased irisin during exercise (4 weeks) after myocardial infarction with ERK-1/2 phosphorylation has been shown to reduce ventricular dilatation, infarction size, fibrosis, cardiomyocyte apoptosis, and increased angiogenesis. While the effects of irisin are blocked by the ERK-1/2 inhibitor (Liao et al., 2019). An acute training session has been shown to cause time-dependent transient activation of ERK-1/2 in the heart. This activation peaks after 15 and 30 min. Increased levels of ERK-1/2 are reduced to resting levels after 24 h of exercise. This represents a temporary increase in ERK−1/2, which is useful for counteracting cardiac apoptosis. Hence, mice in the 12-weeks exercise program (50–70% of VO_2max_) showed significant cardiac hypertrophy by activating several MAPK pathways, an effect that gradually diminishes with exercise-induced cardiac hypertrophy (Iemitsu et al., 2006) (Table 1).

Unlike ERK-1/2, JNK and p38 are stress-activated protein kinases (SAPKs) that respond to environmental stresses such as hypoxia, heat, exercise, inflammatory cytokines, and DNA-damaging agents. Mitochondrial JNK is phosphorylated by H_2_O_2_ stimulation, leading to mitochondrial outer membrane permeability and cytochrome c release (Xia et al., 2016) (Figure 1). JNK inhibition protects cardiomyocyte apoptosis under I/R conditions and is associated with increased Bcl-2 and Bcl-xl family members. Therefore, inhibition of p38 activity is suggested as a central mechanism for heart protection (Xia et al., 2016). In untrained mice, JNK activity in myocytes increases immediately after a training session, whereas in trained mice, no JNK activation is observed above baseline. Therefore, exercise (60–80% of VO_2max_) activates the JNK in an intensity-dependent and adaptive manner, and chronic exercise reverses the myocardial JNK (Boluyt et al., 2003) (Table 1). In mice cardiac muscle, higher levels of phosphorylated p38 have been reported immediately after a treadmill run than before and 1 hour after training (48–60% of VO_2max_). This temporary increase seems to be due to the initial adaptation to exercise (Ludlow et al., 2017). The myocardium of obese rats with a high-fat diet increases phosphorylation levels of P38, mTOR, P70S6k, and 4EBP1 proteins after 2 months of physical exercise (50–55% of VO_2max_). P38 appears to be involved in mTOR activation and this time has not been sufficient for compatibility (Pieri et al., 2014) (Table 1). In the endoplasmic reticulum, exercise reduces inflammatory factors such as p38/JNK and NF-κB. Exercise then regulates the expression of caspase-1, IL-1β, and IL-6, leading to a decrease in caspase-12 and an increase in Bcl-2 in cardiovascular disease (Hong et al., 2017). In rats, aging induces apoptosis in the myocardium by increasing Bax, increasing p-p38, and suppressing HSP70, while treadmill exercise (35–45% of VO_2max_) decreases aging-induced apoptosis by increasing HSP70 expression and suppressing p-p38 expression in cardiac myocytes (Ko et al., 2013). In rats, glutamine supplementation with exercise (85% of VO_2max_) partially prevents p38 and JNK phosphorylation as well as p53 expression and completely reverses the increase in caspase-3 expression (Lagranha et al., 2007) (Table 1). In general, exercise can increase ERK-1/2 in myocytes and decrease JNK and P38 according to severity, compatibility, and location, leading to a decrease in myocyte apoptosis.

### Integrin/FAK pathway

Integrins are used to maintain cell adhesion, and tissue integrity, and to transmit cellular external mechanical signals into the cell. Integrins require cytosolic kinases such as focal adhesion kinase (FAK) to transmit signals into the cell. Integrins consist of two subunits α and β. Integrin β1 is the dominant β subunit in the heart. Mice lacking β1 integrin grow into adulthood and are more vulnerable to myocardial dysfunction and myocyte apoptosis, indicating a protective role of β1 integrin against apoptosis (Xia et al., 2016). Stretching-induced FAK activation stimulates hypertrophic growth associated with mitochondrial biogenesis through up-regulation of PGC1-α, NRF, and Tfam (Ferreira et al., 2018) (Figure 2). FAK is involved in activating the Ras/Raf/MEK/ERK pathway to cause concentric hypertrophy (Watson and Baar, 2014). RhoA, on the other hand, is activated by β1 integrin under mechanical stress, leading to Akt activation through FAK activation (Rindom and Vissing, 2016). It also seems cardiac adaptation to exercise is regulated by integrins and FAK activation (Ferreira et al., 2018). In rats exercising (74% of VO_2max_) on a treadmill, integrin β1, and α5 are increased in cardiac (Burgess et al., 2002; Manso et al., 2009) (Table 2). In the heart, FAK controls the phosphorylation of AKT/GSK-3β and ERK-1/2, and these signaling pathways lead to cell survival and a hypertrophic response after exercise (Brancaccio et al., 2006) (Table 2). In the hearts of trained pigs (5 days a week for 14 weeks), β1 integrin expression is increased (Muthuchamy et al., 2012). FAK absorbs PI3K into membranes in exercise-induced physiological hypertrophy, leading to increased PIP3 and Akt phosphorylation. Akt then activates mTORC1 (Watson and Baar, 2014) (Figure 1). In addition to cardiac muscle in skeletal muscle, FAK phosphorylation and β1 integrin for aerobic and anaerobic exercise (75–98% VO_2max_ three times a week for 8 weeks) increase immediately after exercise (Wilkinson et al., 2008; Flueck et al., 2011; Li et al., 2013; Graham et al., 2015a; Graham et al., 2015b; Franchi et al., 2018) (Table 2). In exercise, cardiac hypertrophy occurs with a proportional increase in collagen and possibly integrin, leading to a highly functional matrix (Burgess et al., 2001). Finally, FAK plays several roles such as myoblast growth, muscle fiber formation, myogenesis, and hypertrophic and anti-apoptotic signals, which reinforces the importance of FAK signaling during muscle growth and homeostasis. However, due to low exercise studies in cardiac tissue, more studies are needed.

**TABLE 2:** A summary of exercise studies on the factors affecting cardiac apoptosis.

Type of exercise	Intensity and duration	Apoptotic and anti-apoptotic factors	Changes	Reference
Treadmill exercise	74% of VO2max	FAK, integrin β1, integrin α5, AKT, ERK-1/2, PI3K, PIP3, mTORC1	Increase	(Burgess et al., 2001; Burgess et al., 2002; Brancaccio et al., 2006; Wilkinson et al., 2008; Manso et al., 2009; Flueck et al., 2011; Muthuchamy et al., 2012; Li et al., 2013; Watson and Baar, 2014; Graham et al., 2015a; Graham et al., 2015b; Franchi et al., 2018)
Aerobic exercise	56–66% of VO2max for 4 weeks	FSTL1-USP10-Notch1, Notch3, sFLT1	Increase	(Xi et al., 2016; Lu et al., 2021)
Aerobic exercise	75% of maximal metabolic for 12 weeks	Notch1/Akt/eNOS	Increase	Liang et al. (2021)
Aerobic exercise	75% of VO2max for 6 weeks	SIRT1, MnSOD, PGC-1α, HIF-1α, VEGF, catalase	Increase	(Rinaldi et al., 2013; Wen et al., 2019)
Swimming training	48–72% of VO2max for 5 weeks	MuRF-1, FOXO1, FOXO3, p53	Decrease	(Ferrara et al., 2008; Kavazis et al., 2014; Li et al., 2017; Donniacuo et al., 2019)
Aerobic exercise and swimming	48–72% of VO2max for 4 and 5 weeks	miR-222, IGF-1, PI3K, Akt, mTOR, AMPK, p70S6K, ERK-1/2, miR-20a, eNOS, miR-17-3p, Bcl-2	Increase	(Oudit and Penninger, 2009; Ma et al., 2013; Pons et al., 2013; Xu et al., 2016; Lin and Zhao, 2017; Shi et al., 2017; Wang et al., 2017; Mayr et al., 2021)
		PTEN, Bax	Decrease	
Swimming training	70% of Vo2max, 1 h per day for 10 weeks	PHLPP-1, TNF-α, NF-κB	Decrease	(Moc et al., 2015; Xing et al., 2016; Lemoine, Fassas, Ohannesian, Purcell; Bernardo et al., 2018)
Swimming training	58% of VO2max for 4 weeks	GSK-3, Bax	Decrease	(Lajoie et al., 2004b; Massicotte and Béliveau, 2006; Hooper, 2007; Li, 2011)
Endurance exercise	30 min in week	Pim-1, BCL-2	Increase	(Dizon et al., 2013; Penna et al., 2020)
Aerobic exercise	70% VO2max for 14 weeks	ROS, RhoA, ROCK1, ROCK2, p38	Decrease	(Matsumoto et al., 2012; Anaruma et al., 2020; Kanazawa et al., 2020; Liu and Wang, 2020)
Aerobic exercise	70–78% of VO2max for 1 week and 1 day	PKCε, PKCδ, PKCɑ, ERK-1/2, Akt	Increase	(Shen et al., 2011; Naskar et al., 2014)
Aerobic exercise	70–80% of VO2max for 60 min	Akt, PKA, AMPK, PKA-Akt-eNOS, PKC, HSP70	Increase	(Melling et al., 2004; Zhang et al., 2009; Tao et al., 2015; Colombe and Pidoux, 2021)
Interval exercise	50–90% of VO2max for 13 weeks	CaMKIIδ	Decrease	Daniels et al. (2015)
Aerobic exercise	60% of maximal speed for 8 weeks	calcineurin/NFAT	Increase	Oliveira et al. (2009)
Swimming exercise	70% of VO2max for 8 weeks	HSP20, HSP27, HSP40, HSP70, HSP72, HSP90	Increase	(Hamilton et al., 2001; Powers and Demirel, 2001; Melling et al., 2007; Quindry et al., 2007; Burniston, 2009)
Aerobic exercise	72% of VO2MAX	JAK2/STAT3, VEGF	Increase	(Sun et al., 2017; Sun and Mao, 2018)

#### Notch signaling pathway

Notch signaling determines the fate of embryonic cells and adult tissue homeostasis through cell-to-cell interactions. Mammalian cells express five notch ligands (delta-like 1, delta-like 3, delta-like 4, Jagged1, and Jagged2) and four notch receptors (Notch 1–4) (Carey et al., 2007; Xia et al., 2016). Jagged1 and Notch1 are expressed in the adult heart and protect heart tissue against cardiomyopathy, myocardial infarction, pathological hypertrophy, and I-R injury (Cai et al., 2016). In the myocardium, Notch signaling is involved in modulating cardiac survival, cardiac stem cell differentiation, and angiogenesis (Ferrari and Rizzo, 2014). Myocardial Notch1 level is activated after injury and enhances the anti-apoptotic signaling Akt and Bcl-2 and improves heart function after myocardial infarction. In contrast, the removal of Notch1 in cardiomyocytes increases apoptosis after hemodynamic overload and Notch inactivation dramatically exacerbates apoptosis. In addition, preconditioning protects against cardiomyocyte apoptosis by activating PI3K/Akt, Pim-1, and Notch1, and inhibiting p38 (Xia et al., 2016) (Figure 1 and Figure 2) (Table 2). Notch signaling also protects heart tissue through Janus kinase 2 (JAK2)/signal transducer and activator of transcription 3 (STAT3) signaling, MnSOD activation, and ROS reduction against myocardial damage (Cai et al., 2016). In addition, Notch3 overexpression in cardiomyocytes plays a protective role against apoptosis (Xia et al., 2016). While Notch3 deficiency indicates low arterial density and increased oxidative stress. Increased expression of anti-angiogenic receptors such as sFLT1 has been reported in mice lacking Notch3 after exercise. While moderate physical activity increases the coronary arteries and capillary density in wild-type mice and capillary density does not change in mice without Notch3 (Ragot et al., 2016). Increased exercise (56–66% of VO_2max_ for 4 weeks)-induced Follistatin-like protein 1 (FSTL1) secretion through the FSTL1-USP10-Notch1 signaling axis has been reported to inhibit myocardial fibrosis in diabetic rats (Xi et al., 2016; Lu et al., 2021) (Table 2). Aerobic exercise (75% of maximal metabolic for 12 weeks) significantly reduces systolic blood pressure, which is independently correlated with skin capillary density and vascular density in humans. While Notch1/Akt/eNOS signaling obstruction almost completely abolishes the protective effect of aerobic exercise on endothelial progenitor cell function (Liang et al., 2021) (Figure 1). In mice, maternal voluntary exercise (3 weeks) reduces congenital heart defects (CHDs), ROS, oxidative stress, and coronary and capillary defects. In this way, the expression of Notch1 and Gata4 cardiac genes is significantly higher in the fetuses of the exercise group compared to the non-exercise group (Saiyin et al., 2019). In addition, Delta-like-1 (DLL-1) is mainly expressed in arterial endothelial cells and plays an important role in stress responses. Serum DLL-1 levels are significantly higher in patients with chronic heart failure than in healthy individuals, and DLL-1 levels have been significantly associated with diastolic dysfunction, decreased exercise capacity, increased levels of C-reactive protein (CRP), and adverse (Norum et al., 2016). In addition, much lower expression of Notch1, Jagged1, and Dll-1 is observed in the muscles of older men (60–75 years) compared with the muscles of young men (18–25 years). However, resistance training increases the expression of Notch1 and decreases the expression of Dll-1. It seems that some of the benefits of resistance training may be achieved through the Notch signaling pathway (Carey et al., 2007). However, due to few sports studies on this subject, more studies are needed.

#### FOXO pathway

FOXO family consists of four members (FOXO1, FOXO3, FOXO4, and FOXO6) whose activity is inhibited by insulin signaling and growth factor (Webb and Brunet, 2014; Alizadeh Pahlavani, 2022a; Alizadeh Pahlavani, 2022b). FOXO acts as a pro-apoptotic agent through the expression of proteins such as Bim, BAD, Bnip3, FasL, and TRAIL in the apoptotic internal and external pathways. Akt phosphorylates the transcription factors of the FOXO family, leading to their exit from the nucleus and their destruction in the cytosol via the ubiquitin-proteasome pathway (Xia et al., 2016). In *drosophila*, after exercise (2 weeks), SIRT1 appears to inhibit the ability of FOXO3 to induce cell death, leading to an increase in a variety of antioxidant genes, including MnSOD (Wen et al., 2019). Long-term and moderate exercise (75% of VO_2max_ for 6 weeks) before infarction injury has also been shown to increase FOXO3 and its two downstream targets, MnSOD, and catalase, compared to sedentary rats with infarction (Donniacuo et al., 2019). In rats, it seems that exercise (48–72% of VO_2max_ for 8 weeks) first significantly increases the FOXO3 protein in the heart and then increases antioxidant levels such as Mn-SOD and catalase. Subsequently, an exercise-induced increase in SIRT1 in the heart prevents the role of FOXO3 apoptosis (Ferrara et al., 2008) (Table 2). After myocardial infarction, exercise (75% of VO_2max_) activates the SIRT1 and SIRT3 pathways, which reduce cardiac apoptosis and oxidative damage by inhibiting p53 and FOXO3 (Donniacuo et al., 2019). Endurance training (swimming for 5 weeks) also can protect the myocardium by activating the SIRT1 pathway and regulating FOXO1 deacetylation (Li et al., 2017). Doxorubicin (DOX) has been reported to increase skeletal and heart myopathy by increasing MuRF-1, FOXO1, and FOXO3 mRNA expression, while short-term exercise training (75% of VO_2max_) protects against acute Doxorubicin-induced heart toxicity by reducing FOXO. In addition, exercises increase PGC-1α in the heart muscle and are associated with the suppression of FOXO activity. Finally, exercise protects against DOX-induced heart myopathy by reducing FOXO1 and MuRF-1 (Kavazis et al., 2014). In confirmation of this, a study reported that prolonged moderate exercise (75% of VO_2max_ for 6 weeks) increases mRNA levels of PGC-1α, HIF-1α, and VEGF genes, which are involved in mitochondrial biogenesis and angiogenesis, and decreases FOXO3, MuRF-1, and Atrogin-1 mRNA levels. Finally, moderate exercise improves cardiac tissue perfusion and regenerates heart muscle (Rinaldi et al., 2013) (Table 2). Thus exercise through Akt, SIRT3, SIRT1, MnSOD, catalase, PGC-1α, HIF-1α, and VEGF reduces p53, MuRF-1, FOXO1, and FOXO3, then reduces cardiac apoptosis.

### Intracellular apoptosis signaling pathways

#### Phosphatase and tensin homologue (PTEN) pathway

Phosphatase and tensin homolog (PTEN) produce phosphatidylinositol-(4,5)-bisphosphate (PIP2) through phosphatidylinositol-(3,4,5)-trisphosphate (PIP3) dephosphorylation and ultimately inhibit Akt activation (Xia et al., 2016). PTEN acts as a negative regulator of PI3K and plays a role in many diseases such as myocardial hypertrophy, heart failure, and preconditioning (Oudit and Penninger, 2009). PTEN expression in cardiomyocytes leads to caspase-3 activation, Bax, and apoptosis. Conversely, specific deletion of PTEN in the heart or PTEN inactivation reduces myocyte apoptosis (Xia et al., 2016). Aerobic exercise works by increasing the activity of IGF-1, PI3Kα, Akt, p70S6K, and decreasing PTEN to maintain heart structure and function in patients with heart failure. Exercise appears to eliminate the inhibitory effect of PTEN on the IGF-1/PI3K/Akt/p70S6K pathway (Oudit and Penninger, 2009) (Table 2). In the myocardium of diabetic mice is increased PTEN expression and PI3K/Akt expression is inhibited. While after training (swimming training for 5 weeks), PTEN expression decreases in the myocardium of diabetic mice, and the PI3K/Akt pathway and miR-222 induction are activated. Therefore, exercise intervention may protect the myocardium under high glucose by activating miR-222 and the PI3K/Akt pathway (Lin and Zhao, 2017) (Figure 1). In mice, exercise (48–72% of VO_2max_ for 4 weeks) also reduces infarction size by up to 60% by increasing phosphorylation of kinases such as Akt, ERK-1/2, p70S6K, AMPK, and GSK3β while reducing levels of PTEN (Pons et al., 2013) (Table 2). These factors protect the heart against myocardial infarction with survival-supporting signaling pathways, reducing phosphatase, and increasing mitochondrial permeability transition pore (mPTP) opening resistance (Pons et al., 2013). In mice with coronary artery disease, VEGF and PTEN are upregulated, while overexpression of miR-20a with exercise (swimming training for 15 weeks) results in decreased PTEN and increased eNOS and VEGF at both transcriptional and translational levels. MiR-20a has been shown to bind specifically to 3′UTR PTEN and induce myocardial cell survival and proliferation by activating PI3K/Akt (Wang et al., 2017) (Table 2). In rats, swimming increases PI3K protein levels, while PTEN and TSC2 decrease by 37 and 22%, respectively, activating the PI3K/AKT/mTOR signaling pathway (Ma et al., 2013). In murine, swimming exercise-induced increase in miR-17-3p leads to inhibition of PTEN and increased hypertrophy, proliferation, and survival in the myocardium. miR-17-3p acts through the tissue inhibitor of metalloproteinase 3 (TIMP3) and the PTEN-AKT pathway to repair myocardium after I/R injury. However, no binding sites for miR-17-3p were detected in the 3′UTR PTEN, indicating that PTEN is not a direct target of miR-17-3p (Shi et al., 2017). In murine, an aerobic exercise-induced increase of miR-17-3p could prevent pathological myocardial dysfunction by inhibiting PTEN because the MEF2C/miR-17-3p/PTEN pathway acts as a new therapeutic strategy for pathological myocardial dysfunction (Xiao et al., 2016; Zhou and Xiao, 2019; Bo et al., 2021b; Rivas et al., 2021) (Figure 1). On the other hand, increased miR-26a after exercise can negatively regulate PTEN and SMAD1 to protect against cardiac damage in diabetic rats, which prevents apoptosis and myocardial atrophy (Icli et al., 2013; Cai et al., 2019). In patients with CAD, there is evidence that after exercise overexpression of miR-19b reduces H_2_O_2_-induced apoptosis and negatively regulates PTEN at the protein level. The overexpression of miR-19b and then decreased PTEN may be a new treatment for myocardial injury after (I-R) because the downregulation of PTEN increases Bcl-2, decreased Bax, and decreased caspase-3 (Xu et al., 2016; Mayr et al., 2021). Finally, it seems that exercise by increasing PI3K/Akt, miR-17-3p, miR-19b, miR-26a, miR-222, and miR-20a can lead to a decrease in PTEN and further stimulate myocardial hypertrophy and proliferation.

### PH domain leucine-rich repeat protein phosphatase pathway

PH domain leucine-rich repeat protein phosphatase (PHLPP) dephosphorylates and inhibits Akt (Xia et al., 2016). There are two isoforms of PHLPP, called PHLPP-1 and PHLPP-2 (Xing et al., 2016). PHLPP targets include Akt, protein kinase C (PKC), p70S6 kinase 1 (p70S6K), and the RAF/MEK/ERK cascade. Tumor necrosis factor-α (TNF-α) increases PHLPP1 levels in cardiac myocytes through NF-κB transcriptional activity after I/R (Xing et al., 2016; Lemoine, Fassas, Ohannesian, Purcell). PHLPP-1 levels are more in the hearts of older mice than in younger mice and overexpression of PHLPP-1 stops p70S6K and Akt phosphorylation and worsens myocyte apoptosis (Xing et al., 2016). In contrast, the hearts of mice lacking PHLPP-1 increase Akt phosphorylation and decrease infarction size after I/R (Xia et al., 2016) (Figure 1). Mice lacking PHLPP-1 also show excellent response to exercise-induced hypertrophy via the Akt, p70S6Kinase, and ERK pathways. Akt phosphorylation in the hearts of mice lacking PHLPP-1 almost doubles and the physiological hypertrophy resulting from swimming (70% of Vo_2max_) is exacerbated by an increase in heart size and myocyte cell area (Lemoine, Fassas, Ohannesian, Purcell; Moc et al., 2015; Bernardo et al., 2018) (Figure 1). PHLPP-1 knockout mice underwent forced swimming training for 20 days which resulted in physiological hypertrophy of the heart compared to wild-trained mice (Moc et al., 2015; Bernardo et al., 2018) (Table 2). Decreased PHLPP-1 in cardiomyocytes by increased exercise (1 h per day for 10 weeks)-induced insulin sensitivity was also shown to significantly increase Akt phosphorylation in animals because insulin suppressed PHLPP-1 to increase Akt activation. Therefore, in diabetic people, PHLPP-1 could be a promising therapeutic target for patients with ischemic heart disease because cardiac apoptosis and infarction size decrease and cardiac function increases (Xing et al., 2016). On the other hand, the pathophysiological hypertrophy caused by pressure overload decreases through swimming exercise along with increased heart size, increased cardiomyocyte levels, and increased expression of hypertrophic genes. This reduction in pathophysiological hypertrophy is associated with reduced fibrosis and cell death in mice lacking PHLPP-1. Also, the expression of VEGF and increased capillary density is higher in the myocardium without PHLPP-1 because deletion of PHLPP-1 can increase endothelial tube formation (Moc et al., 2015) (Table 2). Mice lacking PHLPP-1 under exercise conditions show marked physiological hypertrophy compared to wild-type mice, which is observed with increased cardiac muscle cells (Lemoine, Fassas, Ohannesian, Purcell). Mice lacking PHLPP-1 under swimming exercise compared with wild-type mice lead to increased heart size, angiogenesis, increased capillary density and myocyte cell area compared with wild-type mice, and reduced pathological hypertrophy after aortic stenosis (Moc, 2015) (Table 2). In general, the exercise by decreasing PHLPP-1 and increasing mediators such as Akt, p70S6Kinase, and ERK can lead to increased heart size, increased angiogenesis, increased capillary density, and increased hypertrophic genes, decreased apoptosis, decreased fibrosis, and decreased cell death.

### Glycogen synthase kinase 3 (GSK-3) pathway

Glycogen synthase kinase 3 (GSK-3) is a serine/threonine kinase consisting of two isoforms GSK-3α and GSK-3β and is phosphorylated by insulin. GSK-3 phosphorylation is facilitated by PKB, leading to enzyme inactivation and increased glycogen synthesis. High glycogen content acts as a regulator of GSK-3 and limits further glycogen accumulation (Lajoie et al., 2004a). Akt phosphorylation provides cardiac protection by preconditioning ischemia and inactivating GSK-3, these two factors reduce the size of infarction by a similar amount. GSK-3β-specific deficiency dramatically inhibits apoptosis after myocardial infarction (MI), and conditional deletion of GSK-3α in the heart reduces the Bax/Bcl-2 ratio and apoptosis (Xia et al., 2016). Hyperglycemia and increased glycogen in diabetic mice are associated with dysregulation of PKB and GSK-3. While 13-weeks swimming significantly reduces cardiac glycogen levels and normalizes PKB and GSK-3 in diabetic rats (Lajoie et al., 2004a) (Table 2). Low-dose copper nanoparticles and exercise (swimming, 90 min, 5 days/4 weeks) also significantly prevent infarction through preconditioning and inhibition of GSK-3β (Sharma et al., 2018). In mice with hypertension under training (swimming, 150 min, 5 days/13 weeks) conditions, PKB phosphorylation is significantly improved and is associated with increased GSK-3β phosphorylation. Bax protein expression in the heart of hypertensive mice under training is associated with increased Bcl-2 protein expression, which allows the Bcl-2/Bax ratio to be maintained (Lajoie, 2003). In the left ventricle of mice with hypertension under exercise (70% of VO_2max_), PKB phosphorylation is significantly increased compared with mice with sedentary hypertension and PKB phosphorylation has significantly correlated with GSK-3β phosphorylation. In addition, the expression of HSP72 and Bcl-2 protein is increased in the left ventricle of mice with hypertension under training, which indicates that the anti-apoptotic mechanism is effective in compensating for the increased expression of Bax pro-apoptotic protein in the myocardium (Lajoie et al., 2004b). In confirmation of these findings was shown that in rats long-term moderate-intensity exercise (58% of VO_2max_ for 4 weeks) can improve myocardial cell anti-apoptotic proteins such as HSP72, Bcl-2, and PKB. While they significantly reduce Bax and GSK-3 apoptotic proteins and inhibit myocardial apoptosis (Lajoie et al., 2004b; Massicotte and Béliveau, 2006; Li, 2011) (Table 2). In other words, exercise-induced heat shock proteins activate the PI3K/AKT pathway, which in turn inhibits GSK-3 (Hooper, 2007) (Figure 1). These data suggest that exercise may protect the heart against the harmful effects of GSK-3 because it temporarily inhibits GSK-3 via the PI3K/AKT pathway. This process protects the myocardium and reduces cardiac apoptosis.

### Pim-1 pathway

There is evidence that Akt1 plays a role in the proliferation of heart stem cells and cardiomyocytes, primarily by activating of serine/threonine-protein kinase-1 (Pim-1)-dependent pathway (Mann and Rosenzweig, 2012). Pim-1 regulates many factors of proliferation, survival, and cell cycle (Bishopric, 2012; Liu et al., 2019). Myocyte survival is modulated by Pim-1, heme oxygenase-1, and hypoxia-inducible factor-1α (HIF-α), which act in concert with the Akt signaling network (Oshima et al., 2008). Pim-1 is an oncoprotein that is overexpressed in the lungs of patients with pulmonary arterial hypertension (PAH) and is involved in cell proliferation by activating the nuclear factor of the activated T cells (NFAT)/STAT3 signaling pathway. Plasma levels of Pim-1 in PAH are higher than in the control group and correlate with traditional PAH markers. Pim-1 levels are an independent predictor of mortality and a promising new biomarker in PAH (Renard et al., 2013; Magne et al., 2015; Vaillancourt et al., 2015). The inhibition of Pim-1 activity by genetic manipulation exacerbates cardiomyocyte apoptosis, while Pim-1 expression significantly reduces infarction size in mice. The specific expression of Pim-1 in the heart increases the levels of Bcl-2 and Bcl-xl proteins, which protect against mitochondrial damage due to oxidative stress and Bid pre-apoptotic protein. Inhibition of Pim-1-dependent apoptosis by post-conditioning supports the important role of Pim-1 in regulating cardiac survival after pathological injury (Penna et al., 2009; Xia et al., 2016; Weeks et al., 2017). In addition, Pim-1 significantly leads to the proliferation of myoblasts and stops the acceleration of apoptosis, indicating that Pim-1 is vital for the survival and enhancement of myoblasts (Liu et al., 2019). Physical activity also activates factors to protect the heart. These factors such as Bad/Bax and Pim-1 also participate in the closure of mitochondrial permeability transition pore (mPTP), which is associated with cardiac protection by preventing the opening of mPTP and reducing mitochondrial swelling (Dizon et al., 2013; Penna et al., 2020) (Table 2). Therefore, exercise (70–86% of VO_2max_) reduces and increases the pre-apoptotic and anti-apoptotic proteins of Bax and BCL-2 in rat hearts (Alizadeh et al., 2017; Pahlavani and Veisi, 2018; Alizadeh Pahavani et al., 2020) (Figure 2). Pim-1 also inhibits the phosphorylation of Smad2 and Smad3, prevents their transfer to the nucleus, and suppresses the expression of TGFβ pathway genes. In addition, Pim-1 maintains telomere length in the heart by inhibiting the phosphorylation of Smad2 and Smad3, as it prevents the suppression of telomerase reverse transcriptase (Ebeid et al., 2021). Since exercise studies on Pim-1 have been reported rarely, and on the other hand, upstream and downstream factors of Pim-1, such as Akt and BCL-2, change with exercise, further studies are needed.

### Small GTPases pathway

Small GTPases are subdivided into subtypes of Rho, Rac, and cell division control protein 42 (Cdc42). Overexpression of Ras homolog family member A (RhoA), a GTPase of the Rho subfamily, stimulates heart failure and mitochondrial apoptosis due to increased Bax. In contrast, the relative increase in RhoA and subsequent activation of pro-survival kinases such as Rho, FAK, PI3K/Akt, and PKD play a protective role against myocyte apoptosis. The beneficial role of RhoA through the removal of endogenous RhoA in the heart is characterized by an exacerbation of I/R damage and activation of caspase-3 and apoptosis (Xia et al., 2016). There is evidence that Rac plays a destructive role in cardiomyocyte apoptosis because the removal of Rac1 leads to a decrease in mitochondrial reactive oxygen species (ROS) as well as myocardial dysfunction. In addition, treatment with a Rac1 inhibitor improves cardiomyocyte apoptosis and heart function in type 2 diabetic rats. In contrast, Rac1 expression in the heart leads to the production of superoxide and activation of caspase, pathological hypertrophy, or myocardial dilatation (Xia et al., 2016). In addition, Rho-associated kinase (ROCK) leads to PTEN activation, which in turn reduces PIP3, PDK, and AKT phosphorylation. ROCK also is capable of negatively modulating mTOR and p70S6K, and both proteins are involved in cell growth and survival (Anaruma et al., 2020). When ROCK1 is expressed in the heart, it automatically activates caspase-3, apoptosis, and myocardial fibrosis *in vivo*. In addition, ROCK1 deficiency inhibits myocyte apoptosis, and increased ROCK1 expression causes apoptosis after pressure overload or pathological hypertrophy. ROCK2 appears to play a similar detrimental role to ROCK1 in apoptosis because ROCK2-specific cardiac knockout reduces oxidative stress, myocyte hypertrophy, and apoptosis after transverse aortic stenosis (Xia et al., 2016). ROCK has been reported to be essential for pathological cardiac hypertrophy and is responsible for transmitting p38 to the nucleus. Thus, ROCK and p38 are active in pathological hypertrophy (Anaruma et al., 2020).

Pathological hypertrophic changes are modulated by stimulation of physiological hypertrophic activation by mechanical overloads such as exercise. Swimming exercise induces pathways such as ERK-1/2 activation and AKT/mTOR while no ROCK protein activation is observed. Thus, the factors activated during swimming are the PI3-K/AKT pathway without the participation of ROCK and p38 (Anaruma et al., 2020) (Table 2). In rats, RhoA, ROCK1, and ROCK2 levels are significantly higher in the exhausting exercise than in the without exercise group. While these factors in the preconditioning exercise are significantly less than the exhausting exercise. In the preconditioning exercise, Bcl-2/Bax ratio is significantly higher than in the exhausting exercise. These findings suggest that preconditioning exercise has a protective effect against myocardial injury and improves heart function in mice. The mechanism of this improvement may be related to the Rho/ROCK pathway (Liu and Wang, 2020). High levels of ROCK2 have been seen in mice with myocardial infarction, whereas ROCK2 is significantly reduced with the post-infarction exercise (8 weeks) (Kanazawa et al., 2020) (Table 2). It seems there is an association between ROS and the RhoA/ROCK pathway because inflammatory atherosclerotic lesions show an increase in ROCK, while administration of a RhoA/ROCK inhibitor reduces atherosclerotic plaque size and apoptosis in rats. RhoA mRNA expression is also reduced in endothelial cells of trained hearts (70% VO_2max_ for 14 weeks) (Matsumoto et al., 2012) (Figures 1,2) (Table 2). In addition, RhoA gene expression levels increase in patients with cardiovascular disease while decreasing with exercise (70% of VO_2max_). Therefore, this pathway may be one of the positive mechanisms of cardiac protection due to exercise (Matsumoto et al., 2007). RhoA and ROCK have been reported to be involved in the pathogenesis of the cardiovascular disease, while 8 weeks of exercise reduces RhoA and ROCK gene expression. Therefore, it indicates the inhibitory effect of exercise on the RhoA/ROCK pathway in mice (Matsumoto et al., 2013). During physical activity (70% of VO_2max_), physical stresses such as shear stress activate mechanical transfer mechanisms in endothelial cells and smooth muscle that bind to small GTPases such as RhoA. They stimulate various pathways such as FAK/integrin, c-Src, PI3K, and AKT. These mechanisms regulate anti-atherogenic genes by modifying calcium management and vascular myogenic response to pressure by promoting anti-apoptotic signals (Kojda and Hambrecht, 2005). In addition, Cdc42, a subfamily of small GTPases, inhibits cardiomyocyte apoptosis. While removal of Cdc42 in the heart exacerbates apoptosis (Xia et al., 2016). Cdc42 also inhibits the growth response of the heart to physiological and pathological stimuli (Maillet et al., 2009). For example, 8 weeks of exercise reduces protein levels and RhoA, Rac1, and Cdc42 mRNA levels in the heart (Yan and Li, 2012; Tao et al., 2015) (Figure 1) (Table 2). Mice lacking Cdc42 experience more exercise-induced cardiac hypertrophy and develop heart failure and sudden death more rapidly than controls. Thus Cdc42 is an anti-hypertrophic mediator that acts on exercise-induced physiological hypertrophy to limit heart growth. In addition, Cdc42 is potentially one of the heart’s protective pathways, while removal of Cdc42 in strenuous exercise leads to arrhythmias and sudden death or increased hypertrophy (Maillet et al., 2009). However, more studies are needed in this area.

### Protein kinase C (PKC) pathway

The family of protein kinase C (PKC) consists of at least 12 isoforms. In cardiomyocytes, PKC consists of three main subgroups: the calcium-dependent group (α, βI, βII, and γ), the calcium-independent group (δ, ε, η, θ, and possibly μ), and the atypical PKC (ζ And τ/λ) (Shen et al., 2011). PKC activation is performed by phosphorylation of PDK1 and mTORC2 (Xia et al., 2016). PKCδ or PKCε cause cardiomyocyte hypertrophy by activating ERK-1/2. In addition, concomitant deficiency of PKCδ and PKCε increases pathological cardiac hypertrophy due to pressure overload by inhibiting ERK-1/2. Lack of overexpression of PKCε has also been shown to induce apoptosis in cardiomyocytes, indicating the importance of PKCε for maintaining cell survival (Xia et al., 2016). In mice, PKC-ε signaling pathways are critical for cardioprotection and Akt may be a downstream factor for resistance to myocardial injury because selective inhibition of PKC-ε inhibits Akt activity and blocks sustained cardiac protection (Zhou et al., 2002). In rats, the expression of several isoforms of PKC changes after repeated exercises (70–78% of VO_2max_ for 1 week and 1 day) in the heart, and inhibition of PKC before exercise reverses the improvement of myocardial infarction (Frasier et al., 2011). In the hearts of mice after one exercise (65–70% of VO_2max_) session is increased activation of PKCε, PKCδ, and PKCɑ. While PKC inhibitors suppress exercise-induced myocardial infarction size reduction. Evidence suggests that PKCɑ acts as a regulator of Ca^2+^ and myocardial contraction in cardiomyocytes (Shen et al., 2011) (Table 2). Mice lacking PKCα show an increase in heart contraction (Xia et al., 2016). In rats, during exercise (70% of VO_2max_ for 5 days) PKC is activated by a slight increase in cytosolic Ca^2+^. Indeed, transient Ca^2+^ exposure limits myocardial infarction through PKC activation (French et al., 2008). In mice, during exercise (swimming exercise for 3 weeks) PKCα phosphorylation is involved in cardiac hypertrophy via ERK-1/2, while PKCε activation has a positive effect on ventricular hypertrophy and acts as protection against apoptosis after pre-conditioning. By quitting exercise, inhibition of PKCα leads to inactivation of ERK-1/2, Akt, and activation of caspase-9, indicating the role of PKCα survival in the heart after exercise. In addition, the exercise leads to the reactivation of PKCα and the downstream Akt and ERK-1/2 pathways for improving cardiac function during pathological hypertrophy (Naskar et al., 2014) (Table 2). In rabbits, activation of adenosine receptors and/or opioids during exercise appears to protect the heart through PKC-dependent mechanisms (Miura et al., 2000; Frasier et al., 2011). Under exercise conditions, ROS induced by preconditioning is likely to regulate PKC activity and inhibit mPTP perforating (Zhou et al., 2015). PKC also is activated directly by exercise by producing ROS without the need to interact with a specific receptor, while concomitant administration of a PKC inhibitor destroys cardiac protection (Penna et al., 2020). Overall, studies show that exercise-induced cardiac protection includes Akt, PKCα, PKCδ, PKCε, and ERK-1/2 (Penna et al., 2020) (Figures 1,2). Therefore, exercise can activate calcium-dependent and calcium-independent PKC, leading to cardiac survival factors.

## Cyclic AMP-Dependent protein kinase (PKA) pathway

**FIGURE 3:**
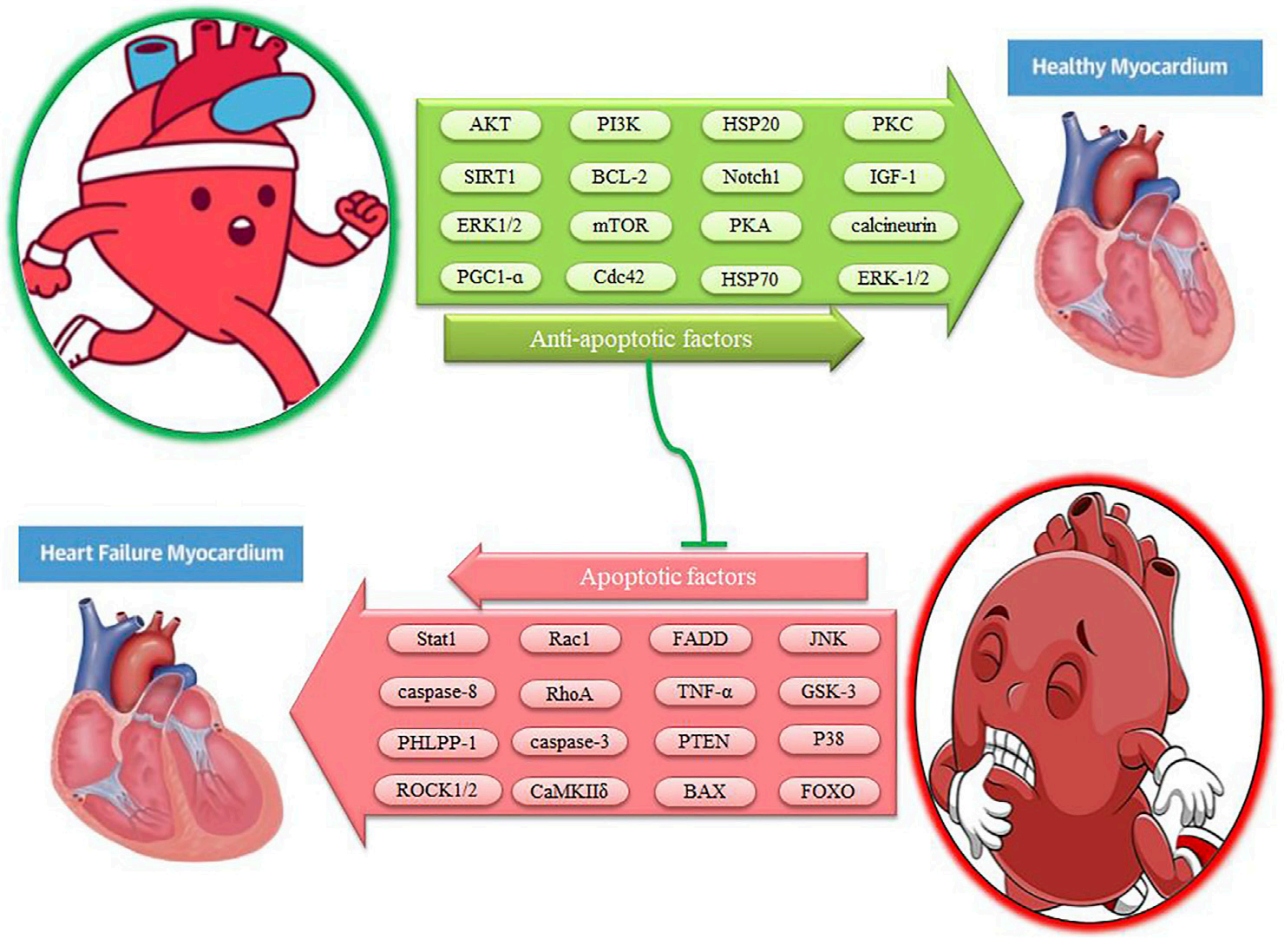
The effect of exercise on myocardial apoptotic factors. Description is available in the text.

Cyclic AMP-dependent protein kinase (PKA) is inactively composed of 2 regulatory subunits and 2 catalytic subunits. With hormone stimulation, the G protein of the α subunit binds to adenylyl cyclase (AC) to convert ATP to cAMP, which then releases and activates PKA catalytic subunits by altering the composition of the regulatory subunits (Xia et al., 2016). In addition, PKA exists in the form of multiple isoforms (RIα, RIβ, RIIα, RIIβ, Ca, Cβ, Cγ, and PRKX). In the heart, isoforms of RIα, RIIα, Ca, and also to a lesser extent Cβ are expressed (Colombe and Pidoux, 2021). It seems decreased PKA activity in the cytoplasm and myofilaments leads to impaired myocardial contraction. In contrast, PKA protective roles have been reported in the heart because PKA activation leads to mitochondrial fusion and cellular protection. Inhibition of apoptosis has been reported to be stopped in the model of diabetic cardiomyopathies with the PKA inhibitor. These findings indicate the role of PKA in the regulation of apoptosis (Xia et al., 2016). Immediately after MI, PKA activity increases heart pump balance and regulates MI-induced cardiac dysfunction. PKA signaling demonstrates cardiac protective effects to limit infarction size by inhibiting Rho-kinase (Colombe and Pidoux, 2021). In rats, high levels of catecholamines in the trained heart (75% of VO_2max_ for 60 min) increase PKA and PKC activity (Melling et al., 2004) (Table 2). In mice, activation of vascular eNOS during exercise (80% of VO_2max_ for 50 min) can be regulated by several signaling pathways including Akt, PKA, and/or AMPK (Zhang et al., 2009; Tao et al., 2015). The PKA-Akt-eNOS pathway reduces harmful heart remodeling and protects heart function (Colombe and Pidoux, 2021). The overall effects of exercise are accompanied by the normalization of myofilament sensitivity to calcium via PKA, as characterized by increased β1-adrenoceptor protein (48%) and cAMP levels (36%) (De Waard et al., 2007). After exercise, PKA phosphorylates stimulation-contraction substrate substrates and modulates chronotropic, inotropic, and lusitropic positive responses (Melling et al., 2006; Colombe and Pidoux, 2021) (Table 2). This process involves phosphorylation of PKA, L-type calcium channels (LTCCs), ryanodine receptor (RyR), and phospholamban (PLB), which increases calcium release and heart rate. In myocardial cells, PKA is the primary regulator of calcium through LTCCs phosphorylation. However, acute PKA activation causes cardiac adaptation (e.g., war or escape response) while chronic activation causes adverse heart regeneration (Colombe and Pidoux, 2021). On the other hand, increased cAMP levels after exercise (70% of VO_2max_) as an upstream PKA activator lead to improved myocardial function during I-R injury. This cardiac protection may be due to cAMP-induced PKA activity that induces HSP70 expression (Melling et al., 2004). PKA plays an essential role in the early regulation of exercise-induced HSP70 gene expression in DNA binding (Melling et al., 2004) (Table 2). Exercise (70% of VO_2max_) induces the expression of the HSP70 gene as the expression of a protective heart protein by activating the heat shock transcription factor (HSF1). While administration of a PKA inhibitor suppresses exercise-induced HSP70 gene expression and demonstrates the role of PKA in regulating HSF1 activation *in vivo* (Melling et al., 2006). HSF1 is also phosphorylated on the two serine residues ERK-1/2 and JNK/SAPK and then prevents myocardial apoptosis (Melling et al., 2006). Exercise increases the frequency of dissolved HSP20 in the heart 2.5-fold, and HSP20 phosphorylation plays a compensatory role in heart disease through PKA/PKG-dependent protein kinases (Fan and Kranias, 2011). Therefore, HSP20 phosphorylation by PKA regulation shows cardiac protection after I/R injury (Colombe and Pidoux, 2021). In addition, PKA regulates many signaling pathways such as ERK-1/2 during exercise (75% of VO_2max_) (Melling et al., 2004) (Figures 1,2, and Figure 3) (Table 2). In general, appears exercise prevents cardiac apoptosis by activating PKA.

### Calcineurin/CaMKII signaling pathway

Calcium signaling regulates several biological processes such as cell survival and death. Intracellular calcium ions (Ca^2+^) bind to calcium-calmodulin (CaM) and activate calcineurin phosphatase. Calcineurin binds to Ca^2+^/CaM with high affinity and is activated with moderate levels of calcium ions, while CaMKII is not very sensitive to calcium ion signals and is activated with high levels of calcium ions (Xia et al., 2016). Calcium/calmodulin-dependent kinases consist of three classes: CaMKI, CaMKII, and CaMKIV. In patients’ myocardium, CaMKII plays a major role in processes such as unfavorable remodeling, arrhythmogenesis, interstitial fibrosis, and apoptosis (Beckendorf et al., 2018). CaMKII is characterized by four isoforms α, β, γ, δ. CaMKIIδ and CaMKIIγ isoforms are present in heart tissue, while the δ isoform is about 2.5 times more important than the isoform CaMKIIγ (Beckendorf et al., 2018). CaMKII activity in human heart failure is up-regulated up to 3-fold and CaMKIIδ expression is increased up to 2-fold (Beckendorf et al., 2018). In the heart, activation of the CaMKIIδC- KB kinase inhibitor (IKK)-NF-κB axis leads to increased expression of TNF-α, and inhibition of IKK or TNF-α is sufficient to reduce the size of myocardial infarction (Beckendorf et al., 2018). CaMKIIδ activity is positively regulated in the myocardium of patients and enhances pathological signals such as hypertrophy, fibrosis, and apoptosis (Daniels et al., 2015). Therefore, excessive CaMKII leads to cardiac pathological hypertrophy, heart failure, and arrhythmia (Bernardo et al., 2018). In contrast, exercise (50–90% of VO_2max_ for 13 weeks) eliminates the negative effects of CaMKIIδ on heart failure because intermittent aerobic exercise reduces CaMKIIδ levels and improves heart function in mice (Daniels et al., 2015) (Table 2). Swimming (6 weeks) may also reverse O-GlcNAcylation-mediated CaMKII activation in type I diabetic mice and ultimately improve heart condition (Bennett et al., 2013; Zhang, 2017). In rats, the expression of CaMKII in the moderate-intensity endurance training (48–60% of VO_2max_ for 6 weeks) group is significantly lower than in the control group and can be effective in some factors in the prevention of cardiovascular disease (Khajehlandi et al., 2020). These results indicate the decreasing of CaMKII as a promising pathway to reduce the progression of heart failure (Kreusser et al., 2014). In addition, CaMKII is also involved in adaptive responses after aerobic exercise and is essential for an adequate cardiac response to a physiological stimulus (Beckendorf et al., 2018). Aerobic exercise (52–78% of VO_2max_) is associated with increased cardiac myocyte contraction, greater calcium amplitude, faster relaxation, PLB phosphorylation, increased SERCA2, and increased activation of CaMKIIδ (Oliveira et al., 2009; Bernardo et al., 2018) (Table 2). Many of these effects are decreased by a CaMKII inhibitor or are decreased in heart failure (Oliveira et al., 2009; Bernardo et al., 2018). Under normal conditions when CaMKIIδ phosphorylation is inhibited, exercise-induced improvements in heart contraction and calcium displacement are reversed and reduced. In this way, CaMKIIδ significantly contributes to cardiomyocyte adaptation to regular exercise (50–90% of VO_2max_) (Kemi et al., 2007) (Figures 1–3). Thus, some of the consequences of CaMKII may be adaptive, while other sustained functions of CaMKII may be detrimental to cardiac integrity and function (Beckendorf et al., 2018). After exercise (endurance swimming training), calmodulin-deficient mice show hypertrophy with increased calcineurin expression, but there are no signs of adverse heart regeneration (Kreusser et al., 2014). Cardiac calcineurin leads to transcription of hypertrophic genes through dephosphorylation of NFAT. Continuous activation of calcineurin protects against cardiac apoptosis, and decreased calcineurin increases apoptosis through an NFAT-dependent mechanism in a model of mice with dilated cardiomyopathy. Thus calcineurin/NFAT is involved in anti-apoptotic settings (Xia et al., 2016). In mice, inactivation of pathological hypertrophy via the calcineurin/NFAT pathway by exercise (60% of maximal speed for 8 weeks) leads to an anti-apoptotic effect on heart failure (Oliveira et al., 2009) (Table 2). In trained mice hearts (59–70% of VO_2max_ for 10 weeks), p-ERK-1/2 and calcineurin increase significantly, leading to physiological left ventricular hypertrophy (Eto et al., 2000; Nakamura et al., 2002). However, there is evidence that physiological hypertrophy of the heart is not affected in response to exercise in NFAT2-deficient mice because, after 4 weeks of voluntary exercise, a cardiac growth response is observed in NFAT2-deficient mice (Bourajjaj et al., 2008). Therefore, exercise can prevent myocardial apoptosis by modulating calcineurin and CaMK and cause physiological cardiac hypertrophy.

### HSPs pathway

Heat shock proteins (HSPs) in cells make up about 5–10% of total proteins, while under stress they increase by up to 15%. HSPs are often found in the cytosol, nucleus, mitochondria, and endoplasmic reticulum (ER), while they are released into the extracellular matrix during cell death or attach freely to the membrane (Wu, Chen, Liu, Liu, Wang, Cheng, et al.). HSPs are involved in maintaining cellular homeostasis and protecting heart cells during periods of acute stress, hypoxia, heat shock, and energy depletion (Powers and Demirel, 2001; Wu, Chen, Liu, Liu, Wang, Cheng, et al.). In rats, HSP20 expression is altered by different models of heart damage, and both HSP20 mRNA and protein levels have been reported to decrease in response to ischemia. While overexpression of HSP20 shows cardiac protective effects (Yin et al., 2019). In the myocardial infarction model, HSP20-regulated exosomes increase cell survival by activating Akt and decreasing mRNA levels of inflammatory factors such as TNF-α and IL-1β. Increased HSP20 expression leads to increased Bcl2 to Bax ratio and inhibition of caspase-3, and myocardial apoptosis (Wu, Chen, Liu, Liu, Wang, Cheng, et al.). In Mice, HSP27 expression is high in mammals after 7 days of MI, whereas it decreases at 14 days after MI. Overexpression of HSP27 increases myocardial infarction tolerance, while HSP27 deficiency exacerbates injury and worsens heart function. Overexpression of HSP27 stops the activation of caspase-3, ROS, the NF-κB-induced inflammatory response, and the degradation of troponin I (cTnI) and cTnT in the myocardium (Wu, Chen, Liu, Liu, Wang, Cheng, et al.; Wang et al., 2019). In rats, cardiac HSP20 is increased with endurance exercise (70–75% of VO_2max_ for 6 weeks), and HSP20 phosphorylation has been identified as a therapeutic target for heart disease (Burniston, 2009) (Table 2). In mice, swimming for 3 weeks also reduces cardiomyocyte apoptosis by inducing extracellular vesicles by activating ERK/HSP27 signaling (Bei et al., 2017). During exercise (65–75% VO_2max_ for 10–12 weeks), HSP72 is important in preventing apoptosis, necrosis, and oxidative damage due to I/R in rat myocytes. HSP72 may help protect cells against a variety of stresses by preventing the accumulation of defective proteins, helping to re-fold damaged proteins (Powers and Demirel, 2001) (Table 2). In rats, after acute exercise (70% of VO_2max_ for 8 weeks), myocardial HSP70 mRNA expression is stimulated in the presence of ERK-1/2 activation. Thus, exercise leads to myocardial adaptation, the onset of proliferative and protective responses (Melling et al., 2007) (Figures 1, 3). Endurance exercise has been shown to increase myocardial HSP72 by 400–500% in young adult rats (Powers and Demirel, 2001). In addition, exercise (70% of VO_2max_) in a warm environment significantly increases myocardial HSP72 content compared to inactive rats. While exercising in a cold environment prevents the increase in HSP72 caused by exercise in the myocardium. However, after myocardial I/R, infarction size and caspase-3 activity decrease in both exercise groups compared with sedentary animals (Quindry et al., 2007) (Table 2). There is evidence that relative levels of HSP90, HSP72, and HSP40 are higher in rats exercising (70% of VO_2max_) in warm water compared with controls and cold water (Hamilton et al., 2001) (Table 2). Therefore, exercise (66% of VO_2max_) in addition to increasing other anti-apoptotic agents, leads to dynamic changes in heat shock proteins to protect the heart that is affected by body temperature (Harris and Starnes, 2001). In general, it seems exercise improves heart function through HSP20, HSP27, HSP40, HSP70, HSP72, HSP90, and other anti-apoptotic agents.

### STATs pathway

Expression of all seven STAT1-7 family members has been reported in cardiomyocytes. Among them, STAT1 and STAT3 have opposite effects on post-ischemic heart cell survival. In the heart of animals under ischemic conditions, STAT1 activation is induced by the production of ROS, which is detrimental to cardiomyocytes through induction of apoptosis and inhibition of autophagy. STAT1 transcription activity is encoded on caspase-1 promoters and also enhances Fas and FasL, which increase cell-level death. In addition, STAT1 induces p53 and Bax transcriptional activity and inhibits Bcl2 (Carroll et al., 2013; Eid et al., 2018). STAT3 is present in most parts of the heart such as the plasma membrane, cytosol, nucleus, and mitochondria. STAT3 is an important cardioprotective agent after ischemia or hypoxia (Eid et al., 2018). STAT3 is activated by a range of cytokines including IL-6 and IL-10, as well as growth factors such as IGF-I, liver growth factor (LGF), epidermal growth factor (EGF), platelet-derived growth factor (PDGF), and Basic fibroblast growth factor (bFGF), ANG II, and mechanical traction (RhoA) (Trenerry et al., 2007; Haghikia et al., 2011). In rats, the cardioprotective effect of STAT3 is mediated by the positive regulation of anti-apoptotic proteins such as BCL-XL, BCL-2, and HSP70. STAT3 also contains the effects of antioxidant proteins such as MnSOD and metallothioneins and enhances angiogenic factors such as VEGF and VE-cadherin. STAT3 prevents the opening of mPTP by activating PI3K, AKT, and ERK-1/2 components and direct regulation of BCL-2. In addition, STAT3 maintains mitochondrial energy and function by improving complex 1 and reducing ROS formation. However, phosphorylation of JAK2 and STAT3 is significantly reduced in human heart failure, which shows an important role in heart regeneration (Eid et al., 2018) (Figures 2, 3). Ischemic preconditioning with exercise in cardiomyocytes activates the JAK/STAT pathway after mechanical traction and pressure overload. This pathway then protects the heart against I/R injury (Boengler et al., 2008; Eid et al., 2018; Zhang et al., 2021). In rats, moderate exercise increases p-JAK2 and p-STAT3 expression and decreases caspase-3 and myocardial apoptosis. While strenuous exercise increases the expression of caspase-3, p-JAK2, and p-STAT3 in the heart (Sun et al., 2017) (Table 2). In addition, after STAT3 phosphorylation, transcription of various hypertrophic factors such as natriuretic peptide type A and natriuretic peptide type B is enhanced (Geng et al., 2019). Rapid and transient activation of STAT3 occurs 2 h after exercise with transient transfer to the nucleus. This process is followed by downstream transcription events such as c-FOS (800-fold), JUNB (38-fold), c-MYC (140-fold), and VEGF (4-fold). Therefore, STAT3 signaling helps to regenerate and adapt after exercise training (Trenerry et al., 2007; Trenerry et al., 2008). In rats, exercise preconditioning (72% of VO_2MAX_) also significantly reduces myocardial ischemic injury through decreased serum cTnI levels, decreased infarction area, decreased myocardial apoptosis, increased Bcl-2 levels, decreased caspase-3 levels, and increased JAK2 and STAT3 phosphorylation. In this way, exercise preconditioning plays a protective role in the heart by activating the JAK2/STAT3 signaling pathway and reducing myocardial cell apoptosis (Sun and Mao, 2018) (Table 2).

## Conclusion

Cardiovascular disease is one of the leading causes of death in the world. Excessive intracellular and extracellular apoptosis is one of the triggers for these deaths. However, several factors, such as aerobic exercise (30–75% of VO_2max_), swimming, short-time (3 days) exercise, and long-time (3 weeks) exercise, can reduce or reverse this process. After exercise, cardiac expression of IGF-1, IGF-1R, p-PI3K, p-Akt, and Bcl2 increases, and these factors also leads to hypertrophy, survival, and cardioprotection (Figures 1–3). After exercise, miR-222, miR-17-3p, MiR-20a, and miR-26a can negatively regulate PTEN to protect the heart against myocardial damage because PTEN inhibits Akt activation and leads to a decrease in Bcl- 2, increases Bax, caspase-3, and apoptosis. Exercise-induced decrease in PHLPP-1 significantly stimulates cardiac hypertrophy through Akt, p70S6Kinase, and ERK, leading to a reduction in pathological hypertrophy, a reduction in cardiac apoptosis, and a reduction in infarct size. The PI3K/AKT pathway then inhibits the GSK-3 apoptotic agent. After exercise, increased anti-apoptotic agents such as Akt, SIRT3, and PGC-1α suppress FOXO activity, which in turn reduces cardiac apoptosis. Exercise can also increase ERK1/2 in myocytes, decrease JNK and P38MAPK, and reduce myocyte apoptosis. During exercise, mechanical stress stimulates β1 and α5 integrins and then activates PI3K/PIP3/Akt/mTORC1 and ERK-1/2 through FAK phosphorylation, leading to cell survival and a hypertrophic response (Figures 1–3). This study shows that the PI3K/Akt pathway is one of the most important pathways to combat apoptosis and protect the heart.

Reduction of exercise-induced apoptosis also occurs through decreased levels of Fas ligand-protein, Fas death receptor, TNF-α, TNFR-1, FADD, caspase-8, and caspase-3. During exercise, also levels of RhoA, ROCK1, Rac1, Cdc42, and ROCK2 are significantly reduced while the Bcl-2/Bax ratio is significantly increased. Under training conditions, PKCε, PKCδ, and PKCɑ are activated, and PKCɑ acts as a Ca^2+^ regulator in the heart and prevents mPTP perforation (Figures 1, 3). During exercise, the PKA-Akt-eNOS pathway protects heart function by inhibiting Rho-kinase and phosphorylation of HSP20 and HSP70 and regulating ERK-1/2 via PKA. Moderate physical activity inhibits myocardial fibrosis by expressing Notch1 cardiac genes, the FSTL1-USP10-Notch1 signaling axis. Exercise also eliminates the negative effects of CaMKIIδ through O-GlcNAcylation on heart failure. Inactivation of exercise-induced pathological hypertrophy via the calcineurin/NFAT pathway leads to an anti-apoptotic effect on heart failure. After acute exercise, expression of Hsp20, HSP27, HSP40, HSP70, HSP72, and HSP90 in the myocardium via JAK2/STAT3 phosphorylation, ERK-1/2 activation leads to myocardial adaptation and proliferative and protective responses (Figure 2, 3). Finally, a variety of exercises block the signaling pathways of intracellular and extracellular apoptosis through various pathways. Since the little study has been done on Pim-1 and FAK, further studies on the effect of exercise on Pim-1 and FAK are needed. Future studies should also try to find other pathways related to cardiac apoptosis. It is also recommended to study the synergistic effect of exercise along with other methods such as gene therapy, dietary supplements, and cell therapy for future research.

## Glossary

${\text{O}}_{2}^{-}$: superoxide anion
**AC**: adenylyl cyclase
**Apaf-1**: apoptosis protease activating factor 1
**bFGF**: Basic fibroblast growth factor
**C/EBPβ**: CCAAT/enhancer-binding protein β
**CaM**: calcium-calmodulin
**cAMP**: cyclic AMP
**Cdc42**: cell division control protein 42
**CHDs**: congenital heart defects
**CRP**: C-reactive protein
**CVDs**: Cardiovascular diseases
**DLL-1**: Delta-like-1
**ECM**: extracellular matrix
**EGF**: epidermal growth factor
**ERK-1/2**: extracellular signal-regulated kinase 1/2
**FADD**: Fas-associated death domain
**FAK**: focal adhesion kinase
**FasL**: Fas ligand
**FSTL1**: Follistatin-like protein 1
**GAPs**: GTPase activators
**GEFs**: guanine exchange factors
**GPCRs**: G protein-coupled receptors
**GSK-3**: glycogen synthase kinase 3
**H2O2**: hydrogen peroxide
**HIF-α**: hypoxia-inducible factor-1α
**HSF1**: heat shock transcription factor
**HSPs**: Heat shock proteins
**IGF-1**: Insulin-like growth factor-1
**IGFI-R**: IGF-I receptor
**IKK**: KB kinase inhibitor
**IKKB**: inhibitor of nuclear factor-kappa B kinase subunit beta
**JAK2**: Janus kinase 2
**JNK**: Jun N-terminal kinases
**LGF**: liver growth factor
**LTCC**: L-type calcium channel
**MAPKs**: Mitogen-activated protein kinases
**MI**: myocardial infarction
**MnSOD**: superoxide dismutase
**mPTP**: mitochondrial permeability transition pore
**MST1**: macrophage stimulating protein 1
**NFAT**: nuclear factor of activated T cells
**Nrf2/Mts**: nuclear factor erythroid 2-related factor/metallothioneins
**p70S6K**: p70S6 kinase 1
**PAH**: pulmonary arterial hypertension
**PDGF**: platelet-derived growth factor
**PHLPP**: PH domain leucine-rich repeat protein phosphatase
**Pim-1**: serine/threonine-protein kinase-1
**PKA**: Cyclic AMP-dependent protein kinase
**PKB/Akt**: protein kinase B
**PKC**: protein kinase C
**PLB**: phospholamban
**PP2C**: protein phosphatase 2C
**PTEN**: phosphatase and tensin homolog
**RhoA**: Ras homolog gene family-A
**ROCK**: Rho-associated protein kinase
**ROS**: reactive oxygen species
**RyR**: ryanodine receptor
**SAPKs**: stress-activated protein kinases
**SERCA2**: sarcoplasmic reticulum Ca2+-ATPase
**SIRT1**: Sirtuin 1
**STAT1**: signal transducer and activator of transcription 1
**STAT3**: signal transducer and activator of transcription 3
**TIMP3**: tissue inhibitor of metalloproteinase 3
**TNFR1**: TNF-α receptor 1
**TNF-α**: tumor necrosis factor-α
**TnI**: troponin I
**TRAIL**: TNF-related apoptosis-inducing ligand
**TRAILR1/2**: TRAIL receptor 1/2
